# Evaluation of human breastmilk adulteration by combining Fourier transform infrared spectroscopy and partial least square modeling

**DOI:** 10.1002/fsn3.1067

**Published:** 2019-05-23

**Authors:** Michele De Luca, Giuseppina Ioele, Claudia Spatari, Luisa Caruso, Maria P. Galasso, Gaetano Ragno

**Affiliations:** ^1^ Department of Pharmacy, Health and Nutritional Sciences University of Calabria Rende Italy; ^2^ Milk Bank "Galatea", Neonatology and Neonatal Intensive Care Unit Cosenza Hospital Cosenza Italy

**Keywords:** adulteration, attenuated total reflection‐Fourier transform infrared, human breastmilk, partial least square, principal component analysis

## Abstract

A two‐step chemometric procedure was developed on the attenuated total reflection‐Fourier transform infrared data of human breastmilk to detect adulteration by water or cow milk. The samples, collected from a Milk Bank, were analyzed before and after adulteration with whole, skimmed, semi‐skimmed cow milk and water. A preliminary clustering via principal component analysis distinguished three classes: pure milk, milk adulterated with water, and milk adulterated with cow milk. A first partial least square‐discriminant analysis (PLS‐DA) classification model was built and then applied on new samples to identify the specific adulterants. The external validation on this model reached 100% of the correct identification of pure milk and 90% of the type of adulterants. In the following step, four PLS calibration models were built to quantify the amount of the adulterant detected in the classification analysis. The prediction performance of these models on new samples showed satisfactory parameters with root mean square error of prediction and percentage relative error lower than 1.38% and 3.31%, respectively.

## 1 INTRODUCTION

Human breastmilk (BM) is the first complete food at birth and considered the natural food par excellence for every newborn: healthy, species‐specific, preventive against allergies, intolerances, and diseases (Georgi, Bartke, Wiens, & Stahl, 2013). The World Health Organization (WHO) recommends breastfeeding for at least the first 6 months of the child's life (Field, 2005). In addition, beside to base nutrients, BM furnishes a large number of biological substances with specific or nonspecific functions that protect the infant against infections and other illnesses (Georgi et al., 2013). The helpful properties of these bioactive molecules are based on their antioxidant and anti‐inflammatory effects, immunostimulatory properties, and bactericidal or bacteriostatic actions on different microorganisms (Goldman & Goldblum, 1995). These defense properties of human BM are particularly suitable for infants of reduced weight and/or preterm infants and in other pathological cases of the newborn. In some circumstances, the infant may not be able to consume milk directly from the mother's breast. In these cases, the previously collected and stored BM should be used (Miranda et al., 2004). The banks of human milk gather, treat, and store milk from healthy lactating women. Human milk banks perform an important social role by promoting breastfeeding and encouraging the mothers to breastfeed their babies. These banks are also an important helper for the care and treatment of premature, low‐weight, sick newborns (Goóes, Torres, Donangelo, & Trugo, 2002).

The commercial interest of BM is becoming a reality in recent years, although in many countries there is no legislation dedicated to this topic. Recent papers have reported cases of infected BM samples or contaminated with cow milk or drug (Keim, Kulkarni, et al., 2015; Keim, McNamara, Kwiek, & Geraghty, 2015). Adulterated BM becomes not only inferior in quality and economic value but it is also dangerous for the infants. The simple addition of water into milk could affect the variation of nutritional composition such as protein and solid content. Infants intolerant to cow milk for allergy could suffer severely if they ingest BM adulterated with this milk (Santos, Pereira‐Filho, & Rodriguez‐Saona, 2013; Zhang et al., 2014).

In the last 25 years, the milk has been studied from many points of view and one of the main topics has been focused on the development of procedures for monitoring quality and safety of the food matrix, from whatever source it comes from (Poonia et al., 2017).

UV and IR spectroscopic methodologies have been widely used for determination of milk adulteration (Kasemsumran, Thanapase, & Kiatsoonthon, 2007). Recio García‐Risco López‐Fandiño Olano and Ramos (2000) have used the capillary electrophoresis for detection of rennet whey solids in milk. Polymerase chain reaction technique has been used to evaluate the milk adulteration due to the mixing of milk from different origins (López‐Calleja et al., 2004). Sandwich ELISA, RP‐HPLC, immune chromatography, and matrix‐assisted laser desorption/ionization time‐of‐flight mass spectrometry (MALDI‐MS) have been used to assay milk adulteration due to soya bean proteins or serum additions (Chávez et al., 2012; Kruså, Torre, & Marina, 2000; De Noo et al., 2005; Oancea, 2009).

The coupling of instrumental analysis with multivariate data analysis techniques, able of handling very large data matrices, is the latest evolution of the methodologies proposed for food analysis (Bassbasi, Luca, Ioele, Oussama, & Ragno, 2014). The chemometric tools applied to the instrumental signals allow to extract the information stored in the data, identifying with remarkable reliability the data patterns and the clustering of the samples (objects), based on the similarities among them. This analytical information can be elaborated for building mathematical models, used to estimate new unknown samples (Bassbasi et al., 2014; Dinç, Ragno, Baleanu, Luca, & Ioele, 2012).

In this perspective, IR spectroscopy shows high sensitivity and specificity and can be used in fingerprint mode analysis, thus becoming a good source of information for multivariate techniques (Rodriguez‐Saona & Allendorf, 2011). Near‐infrared (NIR) spectroscopy and mid‐infrared (MIR) spectroscopy have been widely used for the determination of protein, lactose, and other milk properties (Kawasaki et al., 2008). Since the IR fingerprints show variations in both positions and shapes of the signals in the presence of adulterated milk, some authors have investigated the correlation between NIR and MIR data in the presence of water, whey (Kasemsumran et al., 2007), urea, and caustic soda (Khan, Krishna, Majumder, & Gupta, 2015) by using chemometric analysis (Limm, Karunathilaka, Yakes, & Mossoba, 2018).

Moreover, IR spectroscopy and chemometric procedure are fully adapted to the dictates of the green analytical chemistry (GAC). The role of the analytical chemists should be increasingly focused on developing more environmentally friendly laboratory procedures, by minimizing the use of chemicals, energy consumption, and waste (Gałuszka, Migaszewski, & Namieśnik, 2013; De Luca, Ioele, Spatari, & Ragno, 2017). IR spectroscopy seems appropriate for this purpose as it involves the possibility to analyze complex samples (such as food and environmental matrices) with minimal or no sample preparation, coupled with a simple and fast data collection.

The aim of this work was the development of a multistep analytical procedure for the quality control of BM conferred to milk banks and the assessment of possible adulteration. To our knowledge, to date there are no research works focused on the monitoring of BM adulteration by chemometric techniques. The algorithms principal component analysis (PCA) and partial least square regression (PLS), also as discriminant analysis (PLS‐DA), were used to process the ATR‐FTIR data and design a two‐step procedure. A first discriminant analysis to assess the presence of water or cow milk in the BM samples was followed in the case of a positive response by a quantitative estimate of the amount of adulterant.

## 2 MATERIALS AND METHODS

### 2.1 Calibration and prediction milk samples

Breastmilk samples were collected from the Milk Bank in Cosenza Hospital. The milk is donated by voluntary mothers who respect all the characteristics included in the Bank protocol: health status certification and traceability of donated milk. The cow milk samples were purchased from local dairy product company in Cosenza.

**Figure 1:**
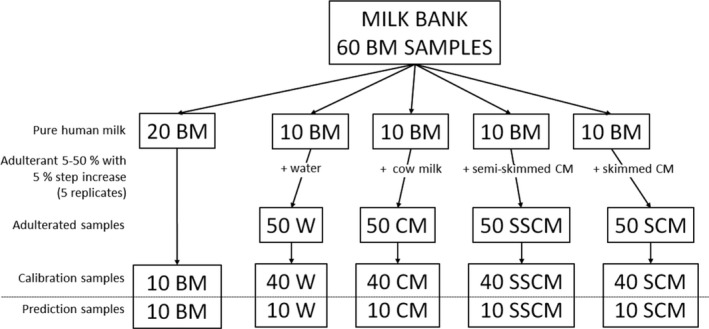
Sample‐making scheme

A set of 220 samples for analysis, consisting of pure BM and adulterated BM, was prepared (Figure 1). For this aim, 60 BM samples (BM set) were made available by the Milk Bank, coming from different mothers donating at different times. These samples were divided into five different groups. A first set of 20 pure BMs was analyzed without any adulteration (set BM), of which 10 samples for modeling and 10 samples for validation procedures, chosen randomly. Fifty samples were prepared from each of the other four groups, adding water (set W), whole cow milk (set CM), semi‐skimmed cow milk (set SSCM), and skimmed cow milk (set SCM), respectively. The amount of adulterant ranged from 5% to 50% with multiple addition of 5%, replicated five times. Applying the Kennard‐Stone sampling method (Galvão et al., 2005), from each set were selected 40 samples for the modeling procedures and 10 samples to validate the models.

### 2.2 Instruments

The IR fingerprints were recorded by using a Spectrum Two Fourier transform infrared (FTIR) spectrometer (Perkin Elmer), equipped with an attenuated total reflection (ATR) accessory consisting of a flat top‐plate fitted with a 25 reflection, 45°, 50 mm ZnSe crystal. The ATR system was cleaned before each analysis by using dry paper and scrubbing it with hexane and ethanol, and spectra acquisition was performed without using cover apparatus. The room air FTIR‐ATR spectrum was used as background to verify the cleanliness and to evaluate the instrumental conditions and room interferences due to H_2_O and CO_2_. FTIR spectra of the milk samples, placed on the ATR surface, were recorded between 4,000 and 450 cm^−1^. Scan number and resolution were optimized at 16 scans and 4 cm^−1^, respectively. The Unscrambler X software version 10.3 from CAMO (Computer Aided Modelling) was used for the chemometric treatment of the spectral data.

### 2.3 Chemometric methods

A PCA study of the data patterns was performed to highlight the differences between samples of pure BM and adulterated BM. The unsupervised data analysis on the milk fingerprints aimed to extract the useful information from the data matrix by projecting samples and variables on a set of new orthogonal variables, called principal components (PCs) (De Luca, Restuccia, Clodoveo, Puoci, & Ragno, 2016; Mabood, Jabeen, Hussain, et al., 2017).

**Figure 2:**
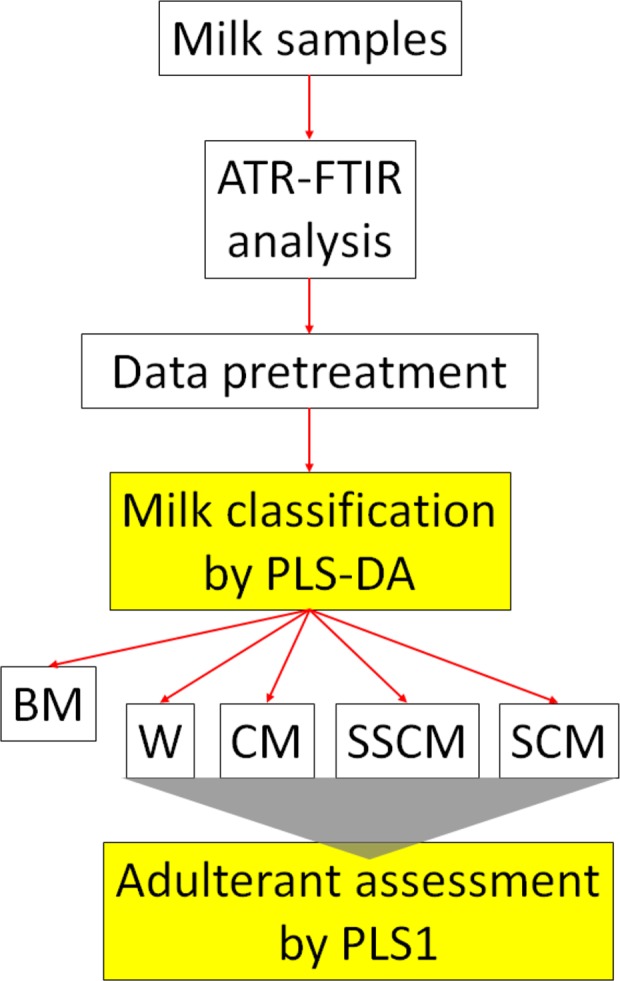
Multistep chemometric procedure

Classification and assessment of adulteration involved the use of various PLS models: a first PLS2 discriminant analysis (PLS‐DA) model designed to detect the possible addition of water or different types of cow milk and subsequent four PLS1 calibration models able of determining the amount of added adulterant. Figure 2 shows the scheme of the analytical procedure.

PLS regression is a factor analysis method, very useful in the processing of spectroscopic data for the calibration analysis of complex samples (Geladi & Kowalski, 1986; De Luca, Ioele, Risoli, & Ragno, 2006; Mabood, Jabeen, Ahmed, et al., 2017; Ragno, Risoli, Ioele, & De Luca, 2006).

In applying PLS procedure, the spectroscopic data (descriptor variables) are arranged in a matrix **X** (*n*,*m*) while a second matrix **Y** contains the concentration data (response variables). The algorithm PLS1 is adopted in the presence of one vector **y**, while PLS2 regression is applied for a matrix **Y** (*n*,*k*) in which the components or classes are more than one (*k* > 1). **X** and **Y** are mean‐centered and then decomposed in factors. Consecutive orthogonal factors are selected with the aim to maximize the covariance between descriptors and responses. PLS modeling is achieved when the factors that explain most of the covariation between both data sets are found (Forina, Oliveri, Lanteri, & Casale, 2008).

In PLS‐DA, Y variable takes on value 1 for the samples belonging to the class and 0 for those not belonging to the class. In calibration, the Y values correspond to the sample composition, and specifically to the amount of milk adulterant in this study (De Luca, Ioele, Spatari, & Ragno, 2016; Palabiyik, Göker, Çaǧlayan, & Onur, 2013). A PLS model can be validated by internal and/or external validation procedure. Full cross‐validation (FCV), a well‐known internal procedure, provided a direct estimate of the error rate incurred by the model. The number of factors was chosen by evaluating the parameters root mean square error of cross‐validation (RMSECV) and correlation coefficient *R* ^2^. The prediction performance of the PLS models was evaluated by an external validation by using new samples (not enclosed in the calibration step). The obtained results were discussed by comparing the figures of merit root mean square error of prediction (RMSEP) and percentage error in predicted concentrations (RE%), calculated as follows:

(1) $$\text{RMSECV or RMSEP}=\sqrt{\frac{\sum_{i=1}^{n}{\left(c_{i}-{\hat{c}}_{i}\right)}^{2}}{n}}$$

(2) $$\text{RE}\left(\%\right)=100\sqrt{\frac{\sum_{i=1}^{n}{\left(c_{i}-{\hat{c}}_{i}\right)}^{2}}{\sum_{i=1}^{n}c_{i}^{2}}}$$

where $c_{i}$ and ${\hat{c}}_{i}$ are, respectively, the known and calculated percentage of milk adulteration in sample *i,* and *n* is the total number of samples in the validation step.

## 3 RESULTS AND DISCUSSION

### 3.1 Exploratory analysis of ATR‐FTIR fingerprints of milk samples

Human and cow milk contain a similar amount of water, but the relative amounts of carbohydrate, protein, fat, vitamins, and minerals vary widely. The protein content in whole cow milk is more than twice that of human milk. The amount of protein in milk is linked to the growth rate of each animal species. Human infant needs less protein and more fat because a large amount of energy is consumed for the development of the brain, spinal cord, and nerves. The proteins in milk consist of two principal categories: caseins and whey. Cow milk contains more casein than human milk, in a ratio of 80:20, whereas in human milk this ratio is 40:60. Whole cow milk and human milk contain a similar amount of fat, but the types of fats are different. Cow milk contains more saturated fat while human milk contains more unsaturated fat. The higher level of unsaturated fatty acids in human milk reflects the important role of these fats in brain growth. In humans, the brain develops rapidly, growing faster than the body and tripling its size in the first year of life. The brain is largely composed of fat and in its development needs a sufficient supply of polyunsaturated essential fatty acids (Chilliard et al., 2014).

**Figure 3:**
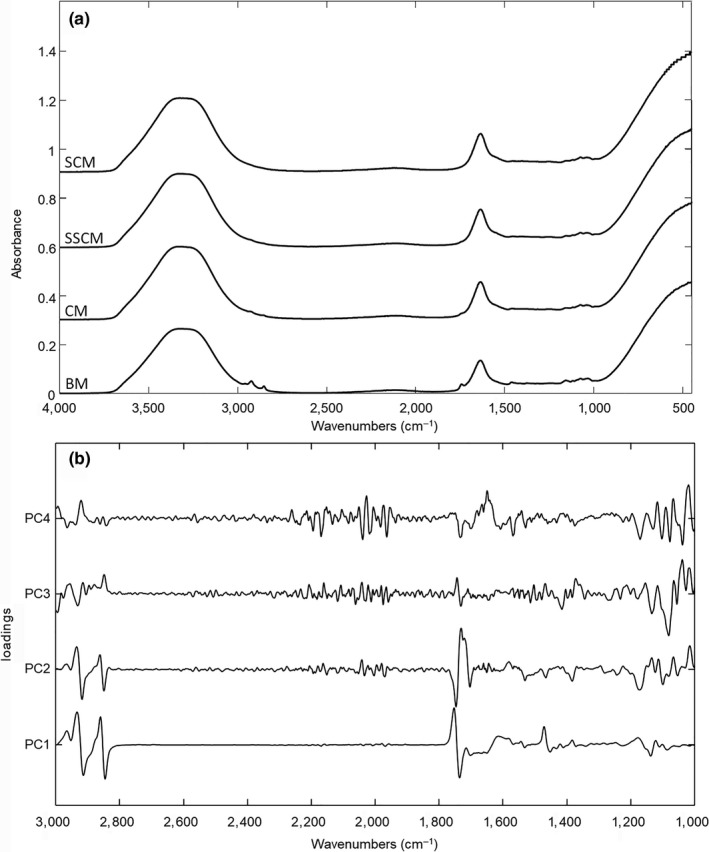
Spectral data: (a) FTIR spectra for pure human breastmilk (BM), whole cow milk (CM), semi‐skimmed cow milk (SSCM), and skimmed cow milk (SCM) samples; (b) Loadings plot for PCs from 1 to 4 resulted from PCA

The FTIR spectra of pure BM and the various types of cow milk are shown in Figure 3a. The absorption bands in the ranges 1,630–1,680 cm^−1^ and 1,510–1,570 cm^−1^ may be associated with C=O stretching or N‐H and C‐H bending vibration of the milk proteins (Carbonaro & Nucara, 2010). The bands 2,920, 2,850, and 1,743 cm^−1^ may be due to the antisymmetric and symmetric stretching of CH_2_ and C=O groups, respectively, from the fatty milk components. The absorption bands in the ranges 3,200–3,700, 1,030–1,200, and 900–450 cm^−1^ have been associated with carbohydrate structures. Absorption bands of water were prominent in regions 3,500–3,000 cm^−1^ and 1,730–1,600 cm^−1^ due to H_2_O stretching and H‐O‐H bending vibration, respectively (Carbonaro & Nucara, 2010).

A very strong overlap between the spectral signals of human milk and pasteurized cow milk is evident throughout the full recorded spectral region, suggesting a high similarity in the composition of the matrices. Therefore, it seemed necessary to perform a multivariate data study to interpret the data matrices, taking into account the full information from the FTIR fingerprints of the samples.

Raw FTIR spectra were pretreated to select the information more useful for the chemometric modeling. First of all, only the wavelength range between 3,000 and 1,000 cm^−1^was considered, discarding the terminal regions because considered rich in instrumental noise and useless information carriers (Kasemsumran et al., 2007). After that, a mathematical pretreatment of the data seemed necessary to minimize instrumental problems as baseline fluctuation or noise. Derivatization by Savitzky–Golay algorithm, standard normal variate (SNV) and multiple scatter correction (MSC), described in several papers, were applied on the FTIR recorded data (Iñón, Garrigues, Garrigues, Molina‐Díaz, & De La Guardia, 2003; De Luca, Ioele, & Ragno, 2014). A significant enrichment in the data variance was reached when the raw data were transformed in derivative signals. Different mathematical parameters were tested in applying the derivative elaboration reaching the best results when the following parameters were set: 1st order, number of smoothing points 7, polynomial order 2.

A PCA explorative analysis of the milk samples was performed on all the FTIR fingerprint spectra previously transformed in derivative data. PCA modeling gave 97.74% of explained variance (EV%), considering the first four PCs. The evaluation of the information available in the PC score plot showed that PC1 (88.35 EV%), PC2 (4.30 EV%), and PC4 (2.09 EV%) were the richest principal components in useful information for our purposes.

Figure 3b shows the loadings values from PCA. The highest values for the first three PCs coincided with the wave ranges 3,000–2,800 cm^−1^, 1,800–1,700 cm^−1^, and 1,200–1,000 cm^−1^, that characterize the different composition of milk samples in terms of proteins, lipids, carbohydrates, and water. Loadings values for PC4 showed a further range rich in information between 1,700 and 1,500 cm^−1^, specific for the protein composition.

**Figure 4:**
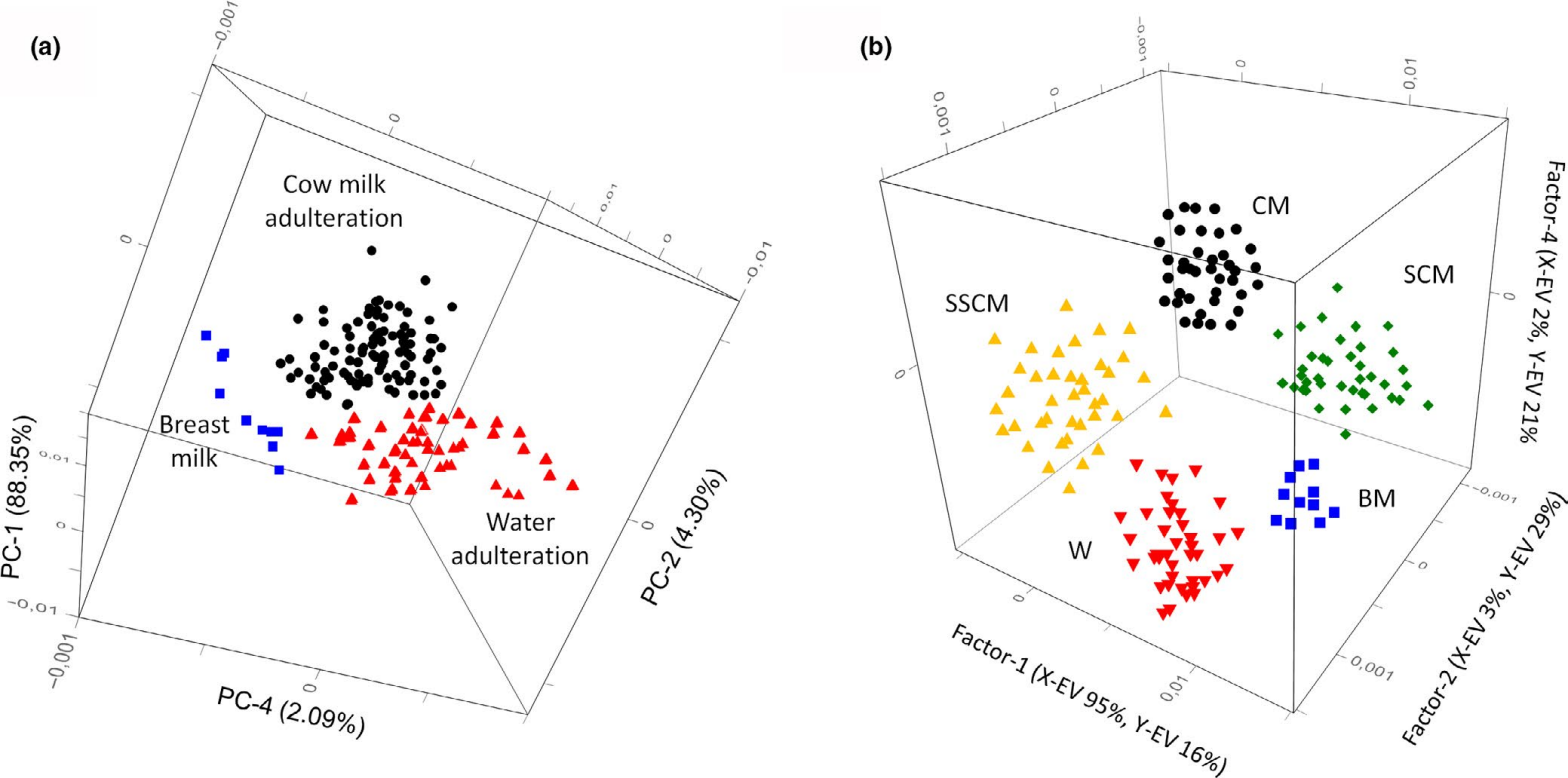
3D score plot (PC1, PC2, and PC4) by PCA (a) and PLS‐DA regression (b)

Figure 4a shows the 3D score plot using PC1, PC2, and PC4. The grouping of the samples was clear making it possible to distinguish the pure BM samples and the BM samples adulterated with water or cow milk. However, this PCA modeling identified only one cluster of BM samples adulterated with cow milk but was unable to distinguish the type of cow milk added.

### 3.2 Classification of milk samples by PLS‐DA modeling

A PLS‐DA modeling was developed with the aim to classify the milk samples as pure BM or adulterated with water (W), whole cow milk (CM), semi‐skimmed (SSCM), and skimmed (SCM). PLS2 algorithm required the setting of more than one Y variable. In this study, five Y variables/classes (Y_BM_, Y_W_, Y_CM_, Y_SSCM_, and Y_SCM_) were set in modeling, assigning the value 1 to the samples belonging to each class and 0 to those belonging to other classes.

The PLS‐DA classification model was validated by full cross‐validation, and its performance evaluated in terms of correlation coefficient *R* ^2^ and RMSECV. The figures of merit, shown in Table 1, were statistically acceptable by considering 6 factors, with RMSECV values between 0.122 and 0.145 and *R* ^2^ in the range 0.857–0.944. Figure 4b shows the score plot factor 2 versus factor 4 by PLS‐DA modeling, in which the discrimination across all classes is greatly improved.

**Table 1:** Statistical parameters of PLS‐DA from FCV procedure

PLS‐DA	Full cross‐validation		
Class Y_n_	Factors	*R* ^2^	RMSECV
BM	6	0.9446	0.1221
W	6	0.9274	0.1379
CM	6	0.8574	0.1432
SSCM	6	0.8718	0.1449
SCM	6	0.9263	0.1231

Abbreviations: BM, breastmilk; CM, cow milk; FCV, full cross‐validation; PLS‐DA, partial least square‐discriminant analysis; RMSECV, root mean square error of cross‐validation; SCM, skimmed cow milk; SSCM, semi‐skimmed cow milk; W, water.

This model was applied to an external prediction set consisting of 60 samples: 10 samples of pure BM and 10 samples from each subset of adulterated samples. According to the PLS‐DA procedure, a sample was considered belonging to a class *n* when the predicted value of Y_n_ was higher than 0.5. The classification results obtained are listed in Table 2 through a confusion matrix. One hundred percent of BM and W samples were well classified while some difficulties were found in classifying the samples adulterated with cow milk. The PLS‐DA model was able to identify the samples adulterated with cow milk, but the exact type of cow milk added was only identified for 90% of the samples. The poor classification of three samples (one SCM and two SSCM) was likely due to the extreme similarity of the BM adulterants. The difference between skimmed and semi‐skimmed milks was due to the lipid composition alone. No samples were detected as suspect origin.

**Table 2:** Confusion matrix from PLS2‐DA external validation

Real					
Y_n_	BM	W	CM	SSCM	SCM
Predicted					
BM	10				
W		10			
CM			10		
SSCM				8	1
SCM				2	9
% CC	100	100	100	80	90

Abbreviations: % CC, % of correct classification; BM, breastmilk; CM, cow milk; PLS‐DA, partial least square‐discriminant analysis; SCM, skimmed cow milk; SSCM, semi‐skimmed cow milk; W, water.

### 3.3 Estimate of adulteration by PLS1 approach

In order to quantify the amount of adulterant added to BM, four PLS1 calibration models were built by using the sets W, CM, SSCM, and SCM, respectively. Full cross‐validation permitted to select the optimal number of factors for all the models by evaluating the parameters *R* ^2^ and RMSECV. The obtained values of the parameters for all the data sets are listed in Table 3.

**Table 3:** Statistical parameters of PLS models from FCV and external validation procedures

PLS	Factors	Full cross‐validation		External validation		
Model		*R* ^2^	RMSECV	*R* ^2^	RMSEP	RE%
PLS_W_	2	0.9884	0.9841	0.9723	1.1718	2.8054
PLS_CM_	3	0.9465	1.3539	0.9267	1.3812	3.3124
PLS_SCM_	4	0.9628	1.3217	0.9616	1.3351	3.1124
PLS_SSCM_	3	0.9822	1.5375	0.9778	1.2155	2.9134

Abbreviations: CM, cow milk; FCV, full cross‐validation; PLS, partial least square; RE%, percentage relative error; RMSECV, root mean square error of cross‐validation; RMSEP, root mean square error of prediction; SCM, skimmed cow milk; SSCM, semi‐skimmed cow milk; W, water.

The calibration report of PLS_W_ described a robust model, since only two factors were enough to explain 98.84% of Y variance. Full cross‐validation showed high prediction ability in determining the amount of water added, providing RMSECV and *R* ^2^ values of 0.988 and 0.9841, respectively.

The three models dedicated to the assay of the different types of added cow milk required three factors for the models PLS_CM_ and PLS_SCM_ and four factors for the PLS_SSCM_ model. The statistical parameters were however satisfactory with *R* ^2^ above 0.947 and RMSECV below 1.54.

The prediction power of the PLS models was tested by performing validation on the external sample set prepared for this aim (see section 2.12.1). Table 3 summarizes the statistical results carried out from the external validation, in terms of *R* ^2^, RMSEP, and percentage relative error (RE%). The prediction procedure showed satisfactory results for all the types of milk adulteration. The model PLS_w_ highlighted the best results with RE% and RMSEP values of 2.81% and 1.172%, respectively. The values of RMSEP were in the range 1.21 and 1.38 in applying the relative PLS models to the samples adulterated with cow milk.

## 4 CONCLUSIONS

The combined use of chemometric methods and IR spectral analysis in a multistep procedure proved to be very effective in detecting even minimal variations in the composition and characteristics of human milk. The handling of ATR‐FTIR spectral fingerprints by PLS regression procedures was able to detect fraudulent additions of water or cow milk into human breastmilk.

In particular, the PLS‐DA technique proved to be particularly robust in discriminating, with a high percentage of correct classification, pure human milk from those adulterated. A further definition of four PLS1 models, specific for the type of adulterant, allowed to determine the amount of adulterant, obtaining excellent results in terms of accuracy and precision when the models were validated on samples external to calibration.

This work demonstrates that ATR‐FTIR spectroscopy has great potential in the control of food matrices whose quality and integrity must be ensured as being of fundamental importance for human health. Moreover, compared to other more complex analytical techniques proposed in the literature, the proposed procedure is inexpensive, requires reduced execution times, and does not require any pretreatment of the samples.

## CONFLICT OF INTEREST

The authors declare that they do not have any conflict of interest.

## ETHICAL APPROVAL

All procedures performed in studies involving human participants were in accordance with the ethical standards of the institutional and/or national research committee and with the 1964 Helsinki declaration and its later amendments or comparable ethical standards.

## INFORMED CONSENT

Informed consent was obtained from all individual participants included in the study.
